# End-of-life care: A retrospective study comparing sarcoma and patients with carcinoma

**DOI:** 10.1177/03008916261446214

**Published:** 2026-06-15

**Authors:** Luca Zambelli, Giacomo Massa, Francesca Ricchini, Ernesto Zecca, Salvatore Provenzano, Gabriele Tinè, Rosalba Miceli, Augusto Caraceni, Paolo Giovanni Casali

**Affiliations:** 1 Palliative Care, Pain Therapy and Rehabilitation Unit, Fondazione IRCCS Istituto Nazionale dei Tumori Milano, Italy; 2 Department of Clinical Sciences and Community Health – Dipartimento di Eccellenza 2023 - 2027, Università degli Studi di Milano, Italy; 3 Medical Oncology Unit 2, Fondazione IRCCS Istituto Nazionale dei Tumori; 4 Biostatistics for Clinical Research, Fondazione IRCCS Istituto Nazionale dei Tumori, Milano, Italy; 5 Department of Oncology and Hematology-Oncology, University of Milan, Milan, Italy

**Keywords:** palliative care, sarcoma, carcinoma, end of life

## Abstract

**Background:**

Soft tissue and bone sarcomas may carry a considerable symptom burden, which typically worsens in the final weeks of life. To help clarify personalized palliative care needs, we investigated discrepancies in end-of-life management between patients with sarcoma and those with the most common carcinomas.

**Patients and methods:**

This retrospective cohort study enrolled all sarcoma patients who died between 1 January 2018 - 31 October 2023, either in the palliative care unit or in medical oncology units, at a large cancer hospital in northern Italy. Each patient with sarcoma was matched with up to three randomly selected patients with carcinoma. Matching was based on age, year of death and unit. Electronic health records were reviewed.

**Results:**

Thirty-six patients with sarcoma were matched with 96 patients with carcinoma (44 lung cancer, 40 breast cancer and 12 colorectal cancer). Patients with sarcoma had a significantly higher chance of palliative sedation compared with patients with carcinoma (OR=32.5). The most common refractory symptom in patients with sarcoma was dyspnea (50%), followed by pain (26%). The average opioid dose difference between patient groups during sedation was as high as 220 mg. The final recorded Richmond Agitation-Sedation Scale score (RASS) was on average -4.4 for patients with sarcoma who died in hospice compared to -2.5 for those who died in an oncology unit.

**Conclusions:**

In our series, patients with sarcoma had a substantially higher risk of experiencing refractory symptoms requiring palliative sedation compared to patients with the most common cancers. Pain and dyspnea were the most common refractory symptoms in patients with sarcoma, resulting in higher dosages of opioids and possibly impacting sedation deepness. This study suggests that patients with sarcoma may pose special challenges to end-of-life care.

## Introduction

Soft tissue and bone sarcomas constitute a heterogeneous group of over 80 different histotypes, collectively representing approximately 1% of all neoplasms.^1^ Surgery, possibly combined with (neo)adjuvant chemotherapy and/or radiotherapy, is the primary treatment for localized sarcomas. However, depending on some prognostic factors, sarcomas carry a significant risk of both local and distant recurrence, the latter often being fatal. Conversely, most metastatic sarcomas cannot be treated with curative intent.^2^ Throughout their illness, patients with sarcoma frequently experience a considerable symptom burden, which typically worsen in the final weeks of life.^3,4^ In the terminal phase, and if symptoms become refractory to conventional palliative care, palliative sedation (PaS) is considered in order to alleviate sufferings. This procedure involves a deliberate proportionate and controlled reduction of consciousness through medications, and sedation depth is continuously monitored to ensure unconsciousness and unresponsiveness to external stimuli.^5,6^

While end-of-life (EoL) for patients with cancer in general has been extensively analyzed, only one study has specifically focused on EoL in patients with sarcoma. One observational retrospective study^7^ reported a higher prevalence of refractory pain and dyspnea (29% and 50%, respectively) compared to cancer patients.^7-9^ Additionally, prior studies have indicated a higher frequency of palliative sedation in patients with sarcoma compared to patients with carcinoma, suggesting a different incidence of refractory symptoms.^10^

This is why we performed this retrospective case series analysis, whose primary objective was to compare the EoL between patients with sarcoma and patients with lung, breast, and colorectal carcinoma who died in either the hospice (HS) or medical oncology (MO) at our institute, which is a tertiary cancer institution and a comprehensive cancer center. Understanding the differences in EoL management and outcomes between sarcoma and patients with carcinoma would allow the identification the actionable factors in order to develop palliative care strategies for patients with sarcoma, with a view to a more personalized palliative care.

## Methods

### Study design

This retrospective matched cohort study was conducted at the Fondazione IRCCS Istituto Nazionale dei Tumori di Milano. This report was written according to Strengthening the Reporting of Observational Studies in Epidemiology (STROBE) Statement. The protocol was approved by the local ethical committee on 24 July 2024 (Comitato Etico Territoriale Lombardia 4, number of protocol INT 174/24).

All patients aged at least 18 years, who died between 1 January 2018 and 31 October 2023 in either HS or in MO units were eligible. All patients with sarcoma were included regardless of primary tumor site, whereas for carcinomas, only patients with lung, breast or colorectal cancer were included.^11^ Participants with more than one cancer diagnoses that could impact the EoL period were excluded.

Due to the larger size of the carcinoma patient sample in comparison with patients with sarcoma, each sarcoma patient was matched with at least one (and up to three) randomly selected carcinoma patient. Given the under-representation of colorectal patients, some patients with sarcoma were matched with multiple carcinoma cases of the same type. Three additional matching criteria were employed: age (⩽40, [40-60], >60 years), year of death (from 2018 to 2023), and unit (HS, MO). Since some patients with sarcoma did not find a perfect match according to all the three aforementioned criteria, matching criteria were relaxed to allow for at least two criteria, and the contiguity between sarcoma and patients with carcinoma in the list served as the third criterion. Patients with sarcoma for which only one matching criterion was met were excluded from the analysis.

### Data collection

Data were retrospectively collected from health patient records. All cases were analyzed from their last hospitalization admission to death.

We collected data on patient demographic and tumor-specific information, including age and gender, tumor histology, primary site, presence and date of local recurrence, presence and date of distant metastases, number of previous oncological treatments, and information regarding last oncological treatment. Additionally, we recorded the date of the last reported Eastern Cooperative Oncology Group (ECOG) Performance Status = 1, the number of admissions within six months before death, the palliative prognostic (PaP) score^12^ without the clinical prediction of survival and the score of End-Of-Life aggressiveness, as per Hui et al. criteria.^13^

PaS was initiated and monitored according to national guidelines^6^ and patterns of PaS were examined, including: the need for PaS; refractory symptom needing PaS; duration of sedation; drug used to maintain deep sedation; level of sedation obtained, using Richmond Agitation & Sedation Scale (RASS^14^); dosages of midazolam and opioids during PaS. Among the refractory symptoms we included dyspnea, delirium, and pain, also considering the possibility of PaS started for acute bleeding or other symptoms. Each symptom was documented based on medical records reported by the medical staff, reflecting patient self- reports. All patients received pharmacological treatments following the World Health Organization (WHO) ladder and international guidelines,^15^ with opioid dose titrated to achieve adequate relief or until dose-limiting toxicity. Maximum opioid use was calculated as from doses of oral morphine based on 24-hour opioid requirements using standardized Oral Morphyne equivalent Daily Dose (OMEDD) factors.^16^ Data were collected independently by L.Z., G.M. and F.R. To minimize possible biases during data collection process, S.P., L.Z., G.M. and F.R independently double-checked extracted data. In case of doubt, a consensus discussion was held among a team of five reviewers (G.M., L.Z., F.R., S.P., E.Z.).

### Statistical analysis

Demographics and EoL features were summarized using descriptive statistics. Specifically, median and interquartile range were used to summarize numerical variables, while absolute and relative frequencies were used for categorical ones. Results were displayed using tables, listings, and/or figures. The difference between sarcoma and other patients with carcinoma according to the reason for the last hospital admission was descriptively explored using contingency tables and descriptive plots.

In order to evaluate whether there was a difference in EoL process between sarcomas and patients with carcinoma, we fitted logistic regression models for binary response variables, multiple linear regressions for normal distributed response variables, and Tweedie regression for zero inflated continuous response variables. Specifically, for initial sedation value of midazolam/opioid dosage and difference between sedation value of midazolam/opioid dosage at the start and at the end of sedation we used Tweedie regression since many patients had dosages equal to 0, as they did not undergo sedation. RASS score at the start or at the end of sedation (⩽ -4 vs > -4), continuous sedation (yes vs no), ECOG at sedation (>3 vs ⩽3), dyspnea as reason for sedation (yes vs no) and number of hospitalizations within six months until death (⩾2 vs 1) were analyzed by means of logistic regression. RASS score was evaluated retrospectively by L.Z., G.M. and F.R. for patients who died in MO. A cut-off as low as -4 was adopted for the RASS score as the distribution was highly skewed toward -5. A similar rationale was applied for the ECOG cut-off, being its distribution skewed toward values higher than 2. Finally, the PaP score value, the time elapsed between the last active therapy and the death, the time elapsed between the last reported ECOG equal to 1 and death and the score of EoL aggressiveness, were analyzed using linear regressions.

In order to compare sarcoma and patients with carcinoma considering the matched nature of the data and other possible unbalancing factors, in all models described above we applied an inverse-probability-weighting (IPW) using a “propensity score”^17^ of being a sarcoma patient.^18^ The latter was estimated by means of a logistic regression model having the type of tumor (sarcomas vs carcinomas) as dependent variable. The logistic model covariates were two of the matching factors (patients’ age as continuous variable and year of death), tumor relapse, number of treatment lines, time elapsed between the diagnosis and death, and number of metastatic sites. The third matching factor was not included in the logistic model as we planned to investigate if the effect of sarcoma and carcinoma group was different for patients treated in HS or MO. To this purpose, all the models included the type of tumor (sarcoma vs carcinoma), the unit and their interaction; the latter was always included in the statistical models as the interest was to explore the direction of effects despite their statistical significance due to the explorative nature of this study. The drawback of maintaining the interaction in all the models is that some models generated extremely wide 95% confidence intervals (CIs) due to the very small number of patients in some subgroups.

Analyses were performed with R (version 4.2.3).

## Results

Thirty-six patients with sarcoma met the inclusion criteria (24 died in HS and 12 in MO unit), 34 of whom could be matched. Matched patients with carcinoma were 96, 44 with lung cancer, 40 with breast cancer and 12 with colorectal cancer. Nineteen patients with sarcoma (55%) had experienced a local relapse, compared to 15 (15%) in the carcinoma group. Patients with sarcoma had a mean of 2.2 metastatic sites, while patients with carcinoma had a mean of 3.9. The most common metastatic site for patients with sarcoma was the lung (16 patients, 47%), while for patients with carcinoma it was lymph nodes (69 patients, 71%). Moreover, 86 patients died in HS (23 sarcoma and 63 patients with carcinoma) and 44 patients died in a MO unit (11 sarcoma and 33 patients with carcinoma). Detailed patient characteristics can be found in Table 1. Online Supplementary Table 1 shows the same characteristics stratified by units and tumor type.

**Table 1.** Patient characteristics.

	Carcinoma	Sarcoma
Total number	96	34
Colon-rectal cancer	12 (12%)	-
Lung cancer	44 (46%)	-
Breast cancer	40 (42%)	-
General characteristics		
Male	27 (29%)	11 (32%)
Female	69 (71%)	23 (68%)
Median age (years)	53	52
Time elapsed from diagnosis to death	26.56 (7.88, 59.99)	24.82 (16.73, 49.74)
Tumor relapsed	40 (42%)	28 (82%)
Local relapse	15 (15%)	19 (55%)
Metastatic disease at diagnosis	57 (59%)	6 (18%)
Number of metastatic sites		
1	2 (2.1%)	14 (41%)
2	13 (14%)	9 (26%)
3	28 (29%)	5 (15%)
4	19 (20%)	3 (8.8%)
5	20 (21%)	2 (5.9%)
⩾6	14 (15%)	1 (2.9%)
Most frequent metastatic site	Lymph nodes (69; 71%)	Lung (16; 47%)
Ward		
Hospice	63 (66%)	23 (68%)
Medical Oncology	33 (34%)	11 (32%)
ECOG pre-sedation		
1	0 (0%)	1 (2.9%)
2	6 (6.3%)	7 (21%)
3	35 (36%)	14 (41%)
4	55 (57%)	12 (35%)

### Patient-related EoL process characteristics

The most common refractory symptom which led to starting PaS in patients with sarcoma was dyspnea (50%, with a large difference between HS 35%, and MO 82%), followed by pain (26%). On the other hand, in patients with carcinoma, delirium was the most common refractory symptom (32%), followed by dyspnea (29%), without major differences between HS and OM (Online Supplementary Table 1). Interestingly, statistical modeling revealed contrasting risks for refractory dyspnea based on cancer type and care setting. In MO, patients with sarcoma had a higher risk of refractory dyspnea (odds ratio – OR=19.01; CI: 1.39-259.4) compared to patients with carcinoma, while in HS the difference was reversed (OR=0.33; CI: 0.11-0.99). Consistently, among patients with lung metastases, patients with sarcoma in HS had a lower risk of developing refractory dyspnea (OR=0.2; CI: 0.06-0.94) compared to patients with carcinoma, while in MO the opposite was true, even if with an extremely wide CI (OR=22.2; CI: 0.75-652). The same analysis was not possible for other refractory symptoms, due to limited numbers (Table 2).

**Table 2.** Patient-related EoL process and sedation-related characteristics.

	Carcinoma	Sarcoma
Continuous sedation	60 (63%)	29 (88%)
(Missing)	1	1
Refractory symptoms requiring sedation		
Dyspnoea	28 (29%)	17 (50%)
Agitation	31 (32%)	4 (12%)
Pain	4 (4.2%)	9 (26%)
Bleeding	0 (0%)	1 (2.9%)
Other	8 (8.3%)	8 (24%)
Reason for no sedation		
Agonic state	1 (1.1%)	0 (0%)
Coma	17 (18%)	0 (0%)
Sudden death	3 (3.2%)	2 (6.0%)
Spontaneous drowsiness	13 (14%)	2 (6.0%)
Resuscitation attempt	1 (1.1%)	0 (0%)
(Missing)	1	1
Midazolam dosage (mg)		
Starting Midazolam dosage (mg)	10.00 (0.00, 15.00)	15.00 (10.00, 24.75)
Final Midazolam dosage (mg)	12.50 (0.00, 25.00)	25.00 (11.25, 37.50)
Number of additional sedative drugs		
0	73 (76%)	17 (50%)
1	18 (19%)	13 (38%)
2	4 (4%)	2 (5%)
3	1 (1%)	1 (3%)
4	0 (0%)	1 (3%)
Type of additional sedative drug		
Promethazine	18 (19%)	14 (41%)
Haloperidol	4 (4%)	4 (12%)
Chloprpromazine	3 (3%)	1 (3%)
Clonidine	2 (2%)	3 (9%)
Propofol	0 (0%)	1 (3%)
Final RASS		
2	0 (0%)	2 (6%)
1	1 (1%)	1 (3%)
0	0 (0%)	0 (0%)
-1	1 (1%)	1 (3%)
-2	1 (1%)	1 (3%)
-3	5 (5%)	4 (12%)
-4	4 (4%)	4 (12%)
-5	83 (86%)	20 (59%)
Missing	1 (1%)	1 (3%)
Opioid dosage (OMEDD)		
Pre-sedation opioid dosage (OMEDD)	120.00 (60.00, 210.00)	120.00 (90.00, 300.00)
(Missing)	32	1
Starting opioid dosage (OMEDD)	45.00 (3.00, 120.00)	120.00 (63.75, 322.50)
Final opioid dosage (OMEDD)	60.00 (3.00, 180.00)	120.00 (63.75, 292.50)

Patients with sarcoma who died in HS had a last reported ECOG PS>3 with a higher frequency (OR=3.8; CI: 1.5-9.3) than patients with carcinoma. Additionally, the trajectory of functional decline differed between the two groups and the two units. The last reported ECOG PS 1 for patients with sarcoma admitted in HS was 2.9 months (CI: 1.2-4.6) farther from death compared to patients with carcinoma, while it was 3.2 months (CI: -6.96-0.49) closer to death compared to patients with carcinoma in MO. Taking into consideration the PaP score without the clinical prediction of survival, HS patients with sarcoma had a higher score (0.92 more; CI: 0.06-1.78) than patients with carcinoma.

There were no time differences from the last oncological treatment administration and death between sarcoma and patients with carcinoma in both units. On the other hand, in HS patients with sarcoma had less chances to undergo at least two emergency hospitalizations during the last six months (OR=0.2; CI: 0.04-0.93) than patients with carcinoma. Patients with sarcoma who died in HS had also a slightly higher score of EoL aggressiveness (0.49 more; CI: 0.25-0.74) than patients with carcinoma.

### Palliative sedation-related characteristics

Palliative sedation (PaS) was deemed necessary for 29 patients with sarcoma (88%) and for 60 patients with carcinoma (63%), with no major differences between units (Online Supplementary Table 1). The statistical model showed that patients with sarcoma had a significantly higher chance of PaS as compared with patients with carcinoma (OR=32.5; CI: 8.68-238). In particular, for patients who died in HS an OR=32.5 (CI: 6.8-155.3) was found, while in MO an OR=10.6 was observed, although with an extremely wide CI.

Baseline opioid use prior to sedation was comparable between groups, with a median OMEDD of 120 mg for both. Notably, MO patients with carcinoma received median half dosage less than patients with sarcoma (Online Supplementary Table 1). In the statistical model, the starting average dose difference in HS between sarcoma and patients with carcinoma was 220 mg (CI: 115-359 mg), compared to 79 mg (CI: -63.5-221.6 mg) for patients dying in MO. Final opioid doses during PaS showed similar trends. Indeed, in HS, patients with sarcoma required 207 mg more OMEDD (95% CI: 102–342 mg) than patients with carcinoma, while in OM the difference was smaller (72.5 mg; 95% CI: -72.5–227.5 mg). Interestingly, among patients who did not receive palliative sedation, there was no difference in the last median OMEDD between groups. However, for those who received palliative sedation, patients with sarcoma’s final median OMEDD was 204 mg higher than that of patients with carcinoma (CI: 84–348 mg). In HS, patients with sarcoma observed a lower opioid dosage increase (difference between last and starting dose) in respect to patients with carcinoma, and this difference decreased at increasing initial dosage; for instance, the increase was of 51.1 mg (CI: 27.7-75.4 mg) less for an initial dosage of 3 mg (1st quartile of the initial dose distribution), of 46.5 mg (CI: 24.8-68.2 mg) less for an initial dosage of 60 mg (median value), of 39.2 mg (CI: 20.5-57.9 mg) less for an initial dosage of 150 mg (3rd quartile).

When considering midazolam starting and final dosages over 24 hours, no significant differences were found between sarcoma and patients with carcinoma using a statistical model, independently of the unit. However, HS patients with advanced sarcoma who started sedation with a midazolam dose higher than 30 mg over 24 hours (3rd quartile) had an increase of 19.8 mg (CI: 10.3-29.3 mg) more than patients with carcinoma during PaS. In addition, patients with sarcoma required on average 1.1 more drugs to maintain deep sedation compared to 0.5 more drugs for patients with carcinoma; clonidine was necessary to achieve deep sedation for three patients with sarcoma and two patients with carcinoma, while for one patient with sarcoma propofol was used with the supervision of an intensive care physician.

Lastly, patients with sarcoma in HS had significantly higher odds of achieving RASS score ⩽ -4 at the initiation of PaS (OR = 10.77; 95% CI: 3.5–32.8), while no differences in the achievement of a final RASS score ⩽4 were found between cancer types or units. Notably, the last reported RASS score was on average -3.8 for sarcoma and -4.7 for patients with carcinoma. For the first group, patients who died in HS had an average RASS of -4.4 compared to -2.5 for those who died in MO unit. Additionally, three patients with sarcoma died with a positive RASS score (2 for two patients and 1 for one patient), compared to one carcinoma patient (RASS score of 1). It was not possible to evaluate these data in a statistical model due to the scarcity of patients with a positive RASS score.

## Discussion

In this retrospective study comparing two case series, respectively, of patients with sarcoma vs patients with carcinoma dying in our institute units, we found that the former had a higher likelihood of requiring PaS compared to the latter, this effect being more pronounced in HS. A higher prevalence of refractory pain and dyspnea was found in patients with sarcoma, while advanced patients with carcinoma more often had general deterioration and organ failures, compromising consciousness and leading to coma. Notably, the prevalence of refractory pain was significantly higher for patients with sarcoma than what is generally reported in cancer patients.^9,19^ Overall, patients with sarcoma received higher doses of opioids compared to patients with carcinoma, again this effect being more pronounced in HS. Interestingly, the difference was statistically significant only for patients requiring sedation due to refractory symptoms. Moreover, even if midazolam daily dose during PaS did not differ across cancer types or medical units, patients with sarcoma required more adjuvant drugs in order to achieve deep sedation. Finally, we found higher RASS scores for patients with sarcoma compared to patients with carcinoma and a greater difference in median RASS score between HS and MO for patients with sarcoma than patients with carcinoma.

This study has important limitations, being retrospective in nature and comparing a limited sample of just three carcinomas, while having the highest incidence worldwide^11^, with a limited sample of patients with sarcoma. Indeed, there was a lack of perfect matching in a 1:3 ratio between sarcoma and patients with carcinoma. The retrospective collection of data from electronical records carries the risk of misinterpretations, especially for data that are not directly specified, such as RASS score for patients who died in MO. Additionally, since we considered only inpatients receiving EoL care, there is a likely bias in favor of those whose EoL care was deemed unsustainable at home with the assistance of palliative homecare services. To our knowledge, however, this is the first study comparing EoL features of sarcoma and patients with carcinoma. As far as sarcomas are concerned, one retrospective study is available, from Kawashima et al., reporting a proportion of refractory pain and dyspnea which was similar to ours (refractory pain = 29% compared to 26% in our series; dyspnea = 50% in both series).^7^

The higher proportion of PaS in patients with sarcoma as compared to those with carcinoma is likely related to the higher proportion of refractory symptoms experienced during EoL, which in turn is related to their peculiar natural history, which is marked by a high proportion of lung metastases, leading to serious dyspnea, and by the fact that sarcomas, which are ubiquitous as far as their site of origin is concerned, can have more variegated extensions, with lesions involving difficult anatomical sites. The higher prevalence of refractory pain and dyspnea in patients with sarcoma is accounted for by the notable differences in opioid dosages during PaS, most often morphine. Interestingly, as for the need for PaS, the difference in opioid dosages during PaS between sarcoma and carcinoma was more marked in HS than in MO. This might be accounted for by a higher number of symptomatic and compromised patients admitted to HS, although it is also true that HS personnel may be especially experienced in managing higher opioid dosages. Notably, during PaS of patients with sarcoma in HS we observed a lower increase in opioid dose and a higher increase in midazolam dose when considering higher starting dosages. This finding can be attributed to the already high baseline dosages of opioids used in HS compared to MO for patients with sarcoma, and to the specific need of obtaining sedation with midazolam, which does not require increasing opioid dose.

Sedation deepness was an issue for some patients with sarcoma, especially for patients who died in MO, even though a palliative care consultation was provided for almost every sarcoma patient (10 out of 11). This can be related to the special expertise needed to manage some sedative drugs. In fact, to achieve deep sedation, patients with sarcoma in some cases needed third/fourth line sedative drugs with a higher sedative power, such as propofol.^6,20^

On the other hand, considering deterioration in patient performance status, those with sarcoma had a shorter time interval than patients with carcinoma between the date of a last reported ECOG PS = 1 and the date of death. This means that, in the period preceding death, their performance status was relatively more preserved. This said, we observed a difference between patients with sarcoma dying at MO and those dying at HS. In fact, patients with sarcoma dying in HS actually exhibited a longer time interval, with a higher probability of pre-sedation ECOG PS>3 in comparison to patients with carcinoma. This corroborates the hypothesis that there is a subset of patients with sarcoma with a higher deterioration rate. As a matter of fact, at our institute they were those selected for admission in HS. However, those with severe life-threatening complications with a relatively preserved PS were preferably admitted to MO.

Finally, patients with sarcoma who died in HS had a higher score of EoL aggressiveness than patients with carcinoma, while having a lower risk of more than two emergency admissions during the previous six months. In particular, they had a substantially higher frequency of hospitalization lasting longer than 14 days during the 30 days before death (21.7% for sarcoma versus 9.5% for patients with carcinoma). It is noteworthy that in the heterogeneity of "sarcoma" histologies, there are some subsets of patients with a higher risk of acute complications, such as colorectal perforation for sarcoma involving the abdomen or bleeding for vascular sarcoma.

In conclusion, although limited in numbers, our case series analysis suggests that EoL care of hospitalized patients with sarcomas poses different challenges compared to more common cancers. Larger series could help to better identify these challenges across the many subtypes of sarcomas, but the difficulty lies in their rarity. Collaborative efforts would thus be welcome, since properly understanding palliative challenges of patients with sarcoma could help tailor their palliative care and improve problematic symptom management. Sarcoma specialists and palliative care specialists should be aware of such challenges affecting such a rare subgroup of cancer patients and be ready to collaborate on a multidisciplinary basis early in their clinical history.

## Supplemental Material

sj-docx-1-tmj-10.1177_03008916261446214 – Supplemental material for End-of-life care: A retrospective study comparing sarcoma and patients with carcinoma Supplemental material, sj-docx-1-tmj-10.1177_03008916261446214 for End-of-life care: A retrospective study comparing sarcoma and patients with carcinoma by Luca Zambelli, Giacomo Massa, Francesca Ricchini, Ernesto Zecca, Salvatore Provenzano, Gabriele Tinè, Rosalba Miceli, Augusto Caraceni and Paolo Giovanni Casali in Tumori Journal
