# Supplementary material for: End-of-life care: A retrospective study comparing sarcoma and patients with carcinoma
Source: Tumori. 2026 Jun 15;112(5):405–12. doi: 10.1177/03008916261446214 (PMC13687600; doi:10.1177/03008916261446214)
Supplement: Supplementary file 1 [file sj-docx-1-tmj-10.1177_03008916261446214.docx]

**Supplementary material**

|  | Hospice (86) | | | Medical Oncology (44) | | |
| --- | --- | --- | --- | --- | --- | --- |
|  | N | Carcinoma (44) | Sarcoma (23) | N | Carcinoma (33) | Sarcoma (11) |
| Continuous sedation | 86 | 38 / 63 (60%) | 19 / 22 (86%) | 44 | 22 / 32 (69%) | 10 / 11 (91%) |
| Missing |  | 0 | 1 |  | 1 | 0 |
| ECOG pre sedation | 86 |  |  | 44 |  |  |
| 1 |  | 0 / 63 (0%) | 0 / 23 (0%) |  | 0 / 33 (0%) | 1 / 11 (9.1%) |
| 2 |  | 1 / 63 (1.6%) | 5 / 23 (22%) |  | 5 / 33 (15%) | 2 / 11 (18%) |
| 3 |  | 18 / 63 (29%) | 9 / 23 (39%) |  | 17 / 33 (52%) | 5 / 11 (45%) |
| 4 |  | 44 / 63 (70%) | 9 / 23 (39%) |  | 11 / 33 (33%) | 3 / 11 (27%) |
| Refractory symptoms requiring sedation |  | | | | | |
| Dyspnoea | 86 | 16 / 63 (25%) | 8 / 23 (35%) | 44 | 12 / 33 (36%) | 9 / 11 (82%) |
| Agitation | 86 | 22 / 63 (35%) | 2 / 23 (8.7%) | 44 | 9 / 33 (27%) | 2 / 11 (18%) |
| Pain | 86 | 3 / 63 (4.8%) | 6 / 23 (26%) | 44 | 1 / 33 (3.0%) | 3 / 11 (27%) |
| Bleeding | 86 | 0 / 63 (0%) | 1 / 23 (4.3%) | 44 | 0 / 33 (0%) | 0 / 11 (0%) |
| Other |  | 86 | 4 / 63 (6.3%) | 8 / 23 (35%) | 44 | 4 / 33 (12%) |
| Midazolam dosage (mg) |  | | | | | |
| Starting Midazolam dosage (mg) | 86 |  |  | 44 |  |  |
| Median (IQR) |  | 10.00 (0.00, 17.50) | 15.00 (15.00, 27.50) |  | 5.00 (0.00, 10.00) | 15.00 (10.00, 15.00) |
| Final Midazolam dosage (mg) | 86 |  |  | 44 |  |  |
| Median (IQR) |  | 15.00 (0.00, 27.50) | 25.00 (15.00, 47.50) |  | 10.00 (0.00, 20.00) | 25.00 (10.00, 30.00) |
| Number of additional sedative drugs | 47 |  |  | 32 |  |  |
| 0 |  | 18 / 37 (49%) | 0 / 10 (0%) |  | 17 / 21 (81%) | 4 / 11 (36%) |
| 1 |  | 14 / 37 (38%) | 6 / 10 (60%) |  | 4 / 21 (19%) | 7 / 11 (64%) |
| 2 |  | 4 / 37 (11%) | 2 / 10 (20%) |  | 0 / 21 (0%) | 0 / 11 (0%) |
| 3 |  | 1 / 37 (2.7%) | 1 / 10 (10%) |  | 0 / 21 (0%) | 0 / 11 (0%) |
| 4 |  | 0 / 37 (0%) | 1 / 10 (10%) |  | 0 / 21 (0%) | 0 / 11 (0%) |
| Missing |  | 26 | 13 |  | 12 | 0 |
| Type of additional sedative drugs | 47 |  |  | 32 |  |  |
| Chloprpromazine |  | 3 / 37 (8.1%) | 1 / 10 (10%) |  | 0 / 21 (0%) | 0 / 11 (0%) |
| Clonidine |  | 2 / 37 (5.4%) | 3 / 10 (30%) |  | 0 / 21 (0%) | 0 / 11 (0%) |
| Propofol |  | 0 / 37 (0%) | 1 / 10 (10%) |  | 0 / 21 (0%) | 0 / 11 (0%) |
| Final RASS | 86 |  |  | 42 |  |  |
| <= -4 |  | 60 / 63 (95%) | 18 / 23 (78%) |  | 27 / 32 (84%) | 6 / 10 (60%) |
| >-4 |  | 3 / 63 (4.8%) | 5 / 23 (22%) |  | 5 / 32 (16%) | 4 / 10 (40%) |
| Missing |  |  |  |  | 1 | 1 |
| Opioid dosage (omedd) |  | | | | | |
| Pre-sedation Opioid dosage (omedd) | 68 |  |  | 29 |  |  |
| Median (IQR) |  | 144.00 (90.00, 249.00) | 135.00 (90.00, 375.00) |  | 60.00 (37.50, 112.50) | 120.00 (67.50, 165.00) |
| Starting opioid dosage (omedd) | 86 |  |  | 44 |  |  |
| Median (IQR) |  | 3.00 (3.00, 195.00) | 120.00 (60.00, 360.00) |  | 60.00 (3.00, 90.00) | 90.00 (82.50, 150.00) |
| Final opiod dosage (omedd) | 86 |  |  | 44 |  |  |
| Median (IQR) |  | 60.00 (3.00, 216.00) | 120.00 (60.00, 360.00) |  | 60.00 (3.00, 120.00) | 120.00 (97.50, 165.00) |

Supplementary Table. *Patient-related EoL process and Sedation-related characteristics split for ward and disease*
